# Maternal smoking during pregnancy and offspring smoking initiation: assessing the role of intrauterine exposure

**DOI:** 10.1111/add.12514

**Published:** 2014-03-17

**Authors:** Amy E Taylor, Laura D Howe, Jon E Heron, Jennifer J Ware, Matthew Hickman, Marcus R Munafò

**Affiliations:** 1 MRC Integrative Epidemiology Unit (IEU) at the University of Bristol, UK Centre for Tobacco and Alcohol Studies, School of Experimental Psychology, University of BristolBristol, UK; 2 MRC Integrative Epidemiology Unit (IEU) at the University of Bristol, School of Social and Community Medicine, University of BristolBristol, UK; 3 Centre for Mental Health, Addiction and Suicide Research, School of Social and Community Medicine, University of BristolBristol, UK

**Keywords:** ALSPAC, intrauterine, maternal smoking, Mendelian randomization, negative control, offspring smoking, pregnancy, tobacco

## Abstract

**Aims:**

To assess whether associations between maternal smoking during pregnancy and offspring smoking initiation are due to intrauterine mechanisms.

**Design:**

Comparison of associations of maternal and partner smoking behaviour during pregnancy with offspring smoking initiation using partner smoking as a negative control (*n* = 6484) and a Mendelian randomization analysis (*n* = 1020), using a genetic variant in the mothers as a proxy for smoking cessation during pregnancy.

**Setting:**

A longitudinal birth cohort in South West England.

**Participants:**

Participants of the Avon Longitudinal Study of Parents and Children (ALSPAC).

**Measurements:**

Smoking status during pregnancy was self-reported by mother and partner in questionnaires administered at pregnancy. Latent classes of offspring smoking initiation (non-smokers, experimenters, late-onset regular smokers and early-onset regular smokers) were previously developed from questionnaires administered at 14–16 years. A genetic variant, rs1051730, was genotyped in the mothers.

**Findings:**

Both mother and partner smoking were similarly positively associated with offspring smoking initiation classes, even after adjustment for confounders. Odds ratios (OR) [95% confidence interval (CI)] for class membership compared with non-smokers were: experimenters: mother OR = 1.33 (95% CI = 1.06, 1.67), partner OR = 1.28 (95% CI = 1.06, 1.55), late-onset regular smokers: mother OR = 1.80 (95% CI = 1.43, 2.26), partner OR = 1.86 (95% CI = 1.52, 2.28) and early-onset regular smokers: mother OR = 2.89 (95% CI = 2.12, 3.94), partner OR = 2.50 (95% CI = 1.85, 3.37). There was no clear evidence for a dose–response effect of either mother or partner smoking heaviness on class membership. Maternal rs1051730 genotype was not clearly associated with offspring smoking initiation class in pre-pregnancy smokers (*P* = 0.35).

**Conclusion:**

The association between smoking during pregnancy and offspring smoking initiation does not appear to operate through intrauterine mechanisms.

## Introduction

Despite the known health risks of smoking, and restrictions on sale and advertising of tobacco products, smoking in adolescence is still common in many countries. In a 2011 survey of UK school children, the prevalence of regular smoking among 15-year-olds was 11% 1. Determining modifiable causes of smoking initiation in adolescence and how initial smoking habits develop into chronic addiction is important for prevention strategies.

It is possible that susceptibility to tobacco dependence may be partly programmed *in utero*, as a result of maternal smoking during pregnancy 2. *In-utero* tobacco exposure may affect the response to early tobacco experimentation in childhood and adolescence, and therefore influence the likelihood of progressing to chronic smoking 3. Maternal smoking during pregnancy is still relatively common; reports suggest that approximately 13% of mothers in England in 2012 were smoking at the time of delivery 4. Therefore, if *in-utero* exposure to tobacco influences offspring smoking, maternal smoking during pregnancy could be a key modifiable risk factor.

There is evidence from animal studies to support an *in-utero* effect of maternal smoking on adolescent smoking behaviour. Prenatal exposure to nicotine has been shown to alter neural cell development in rats, and these changes are still apparent in adolescence 5. Prenatal nicotine exposure is also associated with responses to nicotine and nicotine withdrawal in adolescent rats, including changes in cholinergic synaptic activity and nicotinic acetylcholine receptor regulation and increased nicotine self-administration after withdrawal 6,7. Critically, animal studies have shown that prenatal nicotine exposure influences subsequent offspring sensitivity to nicotine via up-regulation of nicotinic acetylcholine receptors 7,8. In humans, nicotinic receptors are already present in the brain during the first trimester 9, so these effects may operate over a major part of prenatal life. Initial subjective effects of smoking in adolescence are associated with risk of subsequent progression to regular smoking 10, suggesting a possible mechanism through which maternal smoking in pregnancy may influence the risk of offspring smoking. However, while animal models provide evidence for a plausible biological link between nicotine exposure *in utero* and later progression to smoking, the extent to which this evidence is generalizable to human behaviour and biology is unclear.

In humans, evidence from epidemiological studies is inconsistent. Maternal smoking during pregnancy has been linked with increased risk of offspring smoking initiation and tobacco dependence and rates of progression to tobacco dependence 2,11–17. In other studies, associations have attenuated after adjustment for confounders or have been observed only in female offspring 3,18,19. Inferring an intrauterine effect from conventional epidemiological analyses is difficult, because smoking in pregnancy and adolescence have become strongly socio-demographically patterned 20–22, and observed associations are likely to be subject to residual confounding.

Using data from the Avon Longitudinal Study of Parents and Children (ALSPAC), we investigated whether associations of maternal smoking during pregnancy and offspring smoking initiation patterns might be due to intrauterine effects. To do this, we used several strategies for estimating the potential role of non-intrauterine mechanisms in directly observed epidemiological associations. First, we compared associations of mothers’ and mothers’ partners’ smoking during pregnancy with offspring smoking initiation, using partner smoking as a negative control 23. If maternal smoking is influencing offspring smoking initiation via an intrauterine effect, we would expect associations between maternal smoking during pregnancy and offspring smoking initiation to be stronger than associations with partner smoking. We then looked for a dose–response by smoking heaviness and examined associations between postnatal smoking and offspring smoking initiation. If there were an intrauterine effect, we would expect to see a dose–response between smoking heaviness during pregnancy and likelihood of offspring starting to smoke, and stronger associations with offspring smoking patterns in women who continued to smoke during pregnancy than in those who did not smoke during pregnancy but smoked postnatally. These techniques have been applied previously to investigate the effects of pregnancy smoking on offspring birth weight, blood pressure, trajectories of height and adiposity and attention deficit hyperactivity disorder 23–26. One caveat of these methodologies when considering adolescent smoking behaviour as the outcome is that if we observe differences between maternal and partner smoking, or by smoking heaviness, it is possible that these could be attributed to differential influences of these exposures in childhood and adolescence as well as *in utero*. However, our focus is to investigate potential differences observed related to smoking behaviour at the time of pregnancy.

As an additional exploratory analysis, we performed a Mendelian randomization analysis, using a genetic variant associated with smoking behaviour as a proxy for smoking during pregnancy. Because genetic variants are assorted randomly during gamete formation and conception, they should be unrelated to confounding factors and can therefore be used to estimate causal associations free from confounding 27. A single nucleotide polymorphism (SNP), rs1051730, located in the *CHRNA5–CHRNA3–CHRNB4* nicotinic acetylcholine receptor gene cluster (chromosome 15q25) is robustly associated with smoking heaviness, with each copy of the minor allele associated with smoking one additional cigarette per day in smokers 28–30. This variant has also been shown to be a suitable instrument for smoking cessation; in ALSPAC, the minor allele was associated with a 30% increase in the odds of continuing to smoke during pregnancy in women who smoked pre-pregnancy 31. Therefore, if there were an intrauterine effect of maternal smoking on offspring smoking initiation, we would expect to see an association between maternal rs1051730 and offspring smoking initiation in pre-pregnancy smokers. As this variant affects smoking heaviness for as long as an individual smokes, an association could also indicate an effect of maternal smoking during childhood. To test the assumption of no pleiotropy (i.e. that there is no effect of maternal rs1051730 genotype on offspring smoking other than through tobacco exposure) we also performed these analyses in mothers who did not smoke prior to pregnancy.

## Methods

### Study population

ALSPAC is a prospective cohort study which recruited pregnant women residing in Avon, UK, with expected dates of delivery between 1 April 1991 and 31 December 1992. Full details of the study recruitment and methodology have been published previously 32,33. A total of 14 541 pregnancies were included in the initial sample, resulting in 14 062 live births and 13 988 children who were alive at 1 year of age. Detailed information on mothers and their partners (during and after pregnancy) and the children (since birth) has been collected from self-report questionnaires and attendance at clinics. Ethics approval for the study was obtained from the ALSPAC Ethics and Law Committee and the Local Research Ethics Committee.

Please note that the study website contains details of all the data that are available through a fully searchable data dictionary (http://www.bristol.ac.uk/alspac/researchers/data-access/data-dictionary/).

### Parental smoking during pregnancy

Information on mother’s smoking status before, during and after pregnancy was collected in questionnaires administered at 18 and 32 weeks’ gestation and 8 weeks after birth. From these data, a dichotomous variable for maternal smoking during pregnancy was derived, which classified mothers reporting any regular cigarette, cigar, pipe or ‘other’ smoking during pregnancy as pregnancy smokers. Mothers who quit smoking during pregnancy were those who reported smoking regularly prior to pregnancy but did not report smoking at any time during pregnancy. Post-pregnancy starters were those who did not report smoking at any time during pregnancy but had started smoking by 8 weeks after birth. Mothers who continued smoking during pregnancy were those who reported smoking regularly prior to pregnancy and reported smoking at one or more time-points during pregnancy. Information on partner’s smoking before and during pregnancy was obtained from self-reports at 18 weeks’ gestation and 8 weeks after birth. Where self-reported data on partner smoking were not available (29% of partners), maternal reports were used. Where both types of data were available, agreement between partner reports and maternal reports was high (>97%).

Smoking heaviness of mother and partner during pregnancy was based on the maximum daily number of cigarettes, pipes, cigars or others smoked during pregnancy. At each time-point, the partner’s report of his own smoking heaviness was used preferentially over the mother’s report, but the mother’s report was used where there were missing data. Smoking heaviness was categorized into a three-level variable [non-smoker, light smoker (<10 smoked per day) and heavy smoker (≥10 smoked per day)].

### Offspring smoking initiation

Latent classes of smoking initiation in adolescence were developed using Mplus software, based on self-reports of smoking behaviour from two postal questionnaires (administered at 14 and 16 years) and a clinic questionnaire on a computer terminal at aged 15 years. From these data, probabilities of membership of the following classes were derived: non-smokers, experimenters, late-onset regular smokers and early-onset regular smokers. Full details of these classes have been published previously 34. In brief, non-smokers reported very little or no smoking, experimenters tended to smoke infrequently (on a monthly basis), late-onset regular smokers were smoking by age 14 and were mostly daily smokers by age 16, and early-onset regular smokers were mostly daily smokers by age 14. Full-information maximum likelihood was used to estimate probabilities of class membership for individuals who responded to at least one of the smoking questionnaires, but were missing data from at least one questionnaire (4284/7322 or 59% of sample) 34.

### Covariates

Variables considered as potential confounders were sex, maternal age, parity, maternal educational attainment, housing tenure and crowding. Information on mother’s highest educational qualification (CSE, vocational, O-level, A-level or degree) was collected at 32 weeks’ gestation. CSE, vocational and O-level were qualifications taken at 16 years and A-levels were examinations taken at 18 years. Information on housing tenure (mortgaged/owned, council rented, private rented, other) and crowding (number of people in household divided by the number of rooms) was collected from a questionnaire administered at 8 weeks’ gestation.

### Genotype

DNA was extracted from blood samples 35. Genotyping of rs1051730 in the ALSPAC mothers was carried out by KBiosciences (Hoddesdon, UK; http://www.kbioscience.co.uk) using fluorescence-based competitive allele-specific polymerase chain reaction (PCR) (KASPar). Full details of the genotyping methods have been published previously 31. There was little evidence for deviation from Hardy–Weinberg equilibrium for this SNP in the ALSPAC mothers 31.

### Statistical analysis

Analyses were conducted in Stata (version 11). There are relatively few siblings in ALSPAC, so analyses were restricted to the first-born twin of a twin pair and, where there were multiple pregnancies from the same mother in the study, a single child (chosen at random) from each mother. This allowed appropriate standard errors to be calculated. Associations between maternal and partner smoking status and heaviness and offspring smoking initiation were assessed by multinomial logistic regression, using probability weightings for membership of each latent class of smoking initiation. For comparisons of mother and partner smoking during pregnancy, analyses included individuals with full data on all confounders and smoking status for both parents and were additionally mutually adjusted for the smoking behaviour of the other parent. *P*-values for the association of parental smoking with offspring smoking initiation classes were calculated using the likelihood ratio test.

Mendelian randomization analyses were restricted to mothers of self-reported European ancestry, to avoid potential problems of population stratification. For the Mendelian randomization analysis of smoking during pregnancy, multinomial logistic regression was conducted in: (i) non-smokers pre-pregnancy, in order to test the pleiotropy assumption; and (ii) pre-pregnancy smokers, using maternal rs1051730 as an instrument for continuing to smoke during pregnancy. Additive genetic models were assumed, so odds ratios (ORs) represent changes per additional risk (minor) allele. As the genetic variant is used as a proxy for continuing to smoke during pregnancy, we also performed similar observational analyses, in the sample of pre-pregnancy smokers of European ancestry with rs1051730 genotype.

## Results

In total, 6484 ALSPAC offspring (46% male) had complete data on all covariates and were included in the main analyses (flowchart provided in the Supporting information, Fig. S1). Compared to the whole ALSPAC cohort, mothers of included individuals were more likely to have been educated to A-level standard or above (44 versus 35%, *P* < 0.001) and own their home (84 versus 73%, *P* < 0.001), were slightly older (29.2 versus 28.0 years, *P* < 0.001) and were less likely to be regular smokers pre-pregnancy (25 versus 34%, *P* < 0.001) (see Supporting information, Table S1).

The prevalence of smoking during pregnancy was 19% among mothers and 32% among mothers’ partners. Both mother and partner smoking during pregnancy were strongly socio-demographically patterned; smoking during pregnancy was associated with lower maternal education, lower maternal age, lower household social class and lower levels of home ownership (see Table 1). Most offspring were classified as non-smokers at age 16 (84%), with 6% classified as experimenters, 8% as late-onset regular smokers and 2% as early-onset regular smokers.

**Table 1:** Distribution of socio-demographic factors by maternal and partner smoking status during pregnancy

	Mother smoked during pregnancy		P-value[Table-fn tf1-1]	Partner smoked during pregnancy		P-value[Table-fn tf1-1]
Yes (n *=* 1219)	No (n *=* 5265)	Yes (n *=* 2082)		No (n *=* 4402)		
n (%)	n (%)	n (%)		n (%)		
Maternal education						
CSE	293 (24)	539 (10)		370 (18)	462 (11)	
Vocational	144 (12)	400 (8)		219 (11)	325 (7)	
O-level	445 (37)	1800 (34)		779 (37)	1466 (33)	
A-level	251 (21)	1496 (28)		494 (24)	1253 (28)	
Degree or above	86 (7)	1030 (20)	<0.001	220 (11)	896 (20)	<0.001
Housing						
Mortgaged/owned	799 (65)	4649 (88)		1516 (73)	3932 (89)	
Private rented	127 (10)	210 (4)		169 (8)	168 (4)	
Council rented	250 (21)	288 (5)		333 (16)	205 (5)	
Other	43 (4)	118 (2)	<0.001	64 (3)	97 (2)	<0.001
Household social class						
1	79 (7)	991 (20)		185 (9)	885 (21)	
2	457 (40)	2325 (46)		829 (43)	1953 (46)	
3 (non-manual)	297 (26)	1196 (24)		530 (27)	963 (23)	
3 (manual)	210 (19)	399 (8)		286 (15)	323 (8)	
4	74 (7)	137 (3)		103 (5)	108 (3)	
5	15 (1)	15 (0.3)	<0.001	16 (1)	14 (0.3)	<0.001
Parity						
0	587 (48)	2486 (47)		966 (46)	2107 (48)	
1	403 (33)	1908 (36)		729 (35)	1582 (36)	
2+	229 (19)	871 (17)	0.05	387 (19)	713 (16)	0.06
Maternal heaviness						
None	0 (0)	5265 (100)		1258 (61)	4007 (91)	
Light smoker	512 (42)	0 (0)		330 (16)	182 (4)	
Heavy smoker	694 (58)	0 (0)		490 (24)	204 (5)	
Partner heaviness						
None	386 (32)	4007 (76)		0 (0)	4393 (100)	
Light smoker	162 (13)	439 (8)		601 (29)	0 (0)	
Heavy smoker	658 (55)	819 (16)		1477 (71)	0 (0)	
	Mean (SD)	Mean (SD)		Mean (SD)	Mean (SD)	
Maternal age (years)	27.7 (4.9)	29.5 (4.4)	<0.001	28.3 (4.9)	29.6 (4.3)	<0.001

*P*-values derived from χ^2^ tests for categorical variables and *t*-tests for continuous variables. SD = standard deviation; CSE = certificate of secondary education; O-level = general certificate of education ordinary level; A-level = general certificate of education advanced level.

There was strong evidence that maternal smoking during pregnancy was associated with increased odds of offspring being experimenters, late-onset regular smokers and early-onset regular smokers compared to non-smokers (Table 2). The magnitude of association increased across the classes of smoking behaviour; in partially adjusted analyses, maternal smoking during pregnancy was associated with a 1.33-fold [95% confidence interval (CI) = 1.06, 1.67] increase in the odds of being an experimenter, a 1.80-fold (95% CI = 1.43, 2.26) increase in the odds of being a late-onset regular smoker and a 2.89-fold (95% CI = 2.12, 3.94) increase in the odds of being an early-onset regular smoker. Adjustment for partner smoking (fully adjusted model) attenuated associations with all three classes, but these associations remained.

**Table 2:** Associations of maternal and partner smoking during pregnancy with offspring smoking initiation (*n* = 6484)

		Class (percentage membership)*^a^*				P-value*^b^*
Non-smokers (84%)	Experimenters (6%)	Late onset (8%)	Early onset (2%)			
OR (95% CI)	OR (95% CI)	OR (95% CI)	OR (95% CI)			
Mother	Unadjusted	1	1.30 (1.05, 1.61)	2.00 (1.61, 2.47)	3.91 (2.94, 5.21)	<0.001
	Partially adjusted	1	1.33 (1.06, 1.67)	1.80 (1.43, 2.26)	2.89 (2.12, 3.94)	<0.001
	Fully adjusted	1	1.23 (0.97, 1.57)	1.49 (1.17, 1.90)	2.27 (1.64, 3.15)	<0.001
Partner	Unadjusted	1	1.28 (1.07, 1.54)	2.04 (1.68, 2.47)	3.31 (2.49, 4.40)	<0.001
	Partially adjusted	1	1.28 (1.06, 1.55)	1.86 (1.52, 2.28)	2.50 (1.85, 3.37)	<0.001
	Fully adjusted	1	1.22 (1.00, 1.49)	1.68 (1.35, 2.07)	1.96 (1.43, 2.70)	<0.001

Partially adjusted model adjusted for sex, maternal age, parity, maternal educational attainment, crowding and housing tenure. Fully adjusted model additionally mutually adjusted for mother or partner smoking. ^a^Percentages in each class of smoking initiation are based on the highest probability of class membership for each individual; ^b^*P*-value for overall association of the exposure with the outcome from Likelihood Ratio Test. CI = confidence interval; OR = odds ratio.

The magnitudes of associations of partner smoking during pregnancy with offspring smoking initiation classes were very similar to those seen with maternal smoking. In partially adjusted analyses, partner smoking was associated with a 1.28-fold (95% CI = 1.06, 1.55) increase in the odds of being an experimenter, a 1.86-fold (95% CI = 1.52, 2.28) increase in the odds of being a late-onset regular smoker and a 2.50-fold (95% CI = 1.85, 3.37) increase in the odds of being an early-onset regular smoker. For each class of offspring smoking initiation, the CIs for associations of maternal and partner smoking overlapped, indicating that there was little statistical evidence for differences. Adjusting for confounders, and mutual adjustment for other parent smoking, attenuated associations in a similar way to that observed for maternal smoking.

In analyses additionally stratified by smoking heaviness during pregnancy (Table 3), there was some indication of a dose–response for partner smoking on offspring smoking initiation, with ORs higher for heavier smokers than for light smokers for membership of all offspring smoker classes. However, statistical evidence for differences between the point estimates for heavy and light smokers was only weak (*P*-values ≥ 0.048). There was little evidence for a dose–response relationship for maternal smoking during pregnancy; point estimates were slightly higher for light smokers than for heavy smokers for offspring membership of experimenter and late-onset regular smoking classes. There was no strong statistical evidence for a difference in point estimates by maternal smoking heaviness for membership of the early-onset regular smoker class.

**Table 3:** Associations of maternal and partner smoking heaviness during pregnancy with offspring smoking initiation (*n* = 6471)

		n	Class (percentage membership)*^a^*				P-value*^b^*
Non-smokers (84%)	Experimenters (6%)		Late onset (8%)	Early onset (2%)			
OR (95% CI)	OR (95% CI)		OR (95% CI)	OR (95% CI)			
Mother	Non-smoker	5265	1	1	1	1	
	Light smoker	512	1	1.51 (1.12, 2.04)	1.94 (1.43, 2.64)	2.51 (1.62, 3.88)	
	Heavy smoker	694	1	1.12 (0.82, 1.52)	1.65 (1.23, 2.21)	3.17 (2.22, 4.53)	<0.001
Partner	Non-smoker	4393	1	1	1	1	
	Light smoker	601	1	1.18 (0.87, 1.60)	1.45 (1.04, 2.01)	1.84 (1.13, 2.98)	
	Heavy smoker	1477	1	1.33 (1.07, 1.65)	2.06 (1.65, 2.57)	2.78 (2.02, 3.82)	<0.001

Adjusted for sex, maternal age, parity, maternal educational attainment, crowding and housing tenure. Light smoking is defined as <10 cigarettes per day, heavy smoking ≥10 cigarettes per day. ^a^Percentages in each class of smoking initiation are based on the highest probability of class membership for each individual; ^b^*P*-value for overall association of the exposure with the outcome from Likelihood Ratio Test. CI = confidence interval; OR = odds ratio.

Odds of offspring being experimenters or late-onset regular smokers compared to non-smokers did not differ according to whether mothers continued to smoked during pregnancy (*n* = 1236) or did not smoke during pregnancy but started soon after birth (*n* = 100) (see Supporting information, Table S2). The odds of offspring being an early-onset smoker compared to a non-smoker were higher for continuing smokers (OR = 2.99; 95% CI = 2.20, 4.08) than women who did not smoke during pregnancy but smoked postnatally (OR = 1.86; 95% CI = 0.66, 5.23) but CIs were wide due to the small number of post-pregnancy starters, so there was no clear statistical evidence for a difference between these groups.

Of the sample of pre-pregnancy smokers included in the Mendelian randomization analysis (*n* = 1020), 28% quit smoking during pregnancy (see Supporting information, Fig. S2 for a flowchart of the analysis sample). In the observational analysis, there was good evidence that continuing to smoke during pregnancy increased the odds of offspring being early-onset regular smokers (adjusted OR = 3.18; 95% CI = 1.32, 7.67) and some evidence that continuing to smoke during pregnancy increased odds of offspring being late-onset regular smokers (adjusted OR = 1.65; 95% CI = 0.97, 2.79) (Table 4). There was no strong evidence that continuing to smoke during pregnancy was associated with the odds of offspring being experimenters (adjusted OR = 1.09; 95% CI = 0.68, 1.76). In Mendelian randomization analysis, there was no strong evidence that maternal rs1051730 genotype was associated with offspring smoking initiation in mothers who were non-smokers pre-pregnancy (*P* = 0.67) or mothers who smoked prior to pregnancy (*P* = 0.35) (Table 4).

**Table 4:** Observational and Mendelian randomization analyses for continuing to smoke during pregnancy in mothers of European ancestry with rs1051730 genotype

	n	Class				P-value*^b^*
Non-smokers		Experimenters	Late onset	Early onset		
OR (95% CI)		OR (95% CI)	OR (95% CI)	OR (95% CI)		
Observational						
Smokers pre-pregnancy (unadjusted)	975	**1**	1.05 (0.66, 1.68)	1.68 (1.01, 2.81)	3.62 (1.53, 8.57)	0.002
Smokers pre-pregnancy (adjusted)^a^	975	**1**	1.09 (0.68, 1.76)	1.65 (0.97, 2.79)	3.18 (1.32, 7.67)	0.01
Mendelian randomization						
Non-smokers pre-pregnancy	3133	1	1.06 (0.88, 1.28)	0.90 (0.72, 1.12)	0.92 (0.63, 1.34)	0.67
Smokers pre-pregnancy	1020	1	0.95 (0.69, 1.30)	1.16 (0.86, 1.57)	1.36 (0.92, 2.00)	0.35

Odds ratios (ORs) for observational analysis represent relative differences of offspring smoking class membership for mothers who continue to smoke during pregnancy compared to those who quit smoking during pregnancy. ORs for Mendelian randomization analysis represent relative differences of offspring smoking class membership per each additional minor (risk) allele of rs1051730. Samples restricted to mothers of European ancestry with rs1051730 genotype data. ^a^Adjusted for sex, maternal age, parity, maternal educational attainment, crowding and housing tenure. ^b^*P*-value for overall association of the exposure with the outcome from Likelihood Ratio Test. CI = confidence interval.

## Discussion

We do not find sufficient evidence to support the hypothesis that the association between maternal smoking and offspring smoking initiation operates through an intrauterine effect. We found similar-sized associations between maternal and partner smoking during pregnancy and offspring smoking initiation patterns, and little evidence for a dose–response relationship between maternal smoking heaviness during pregnancy and offspring smoking initiation. The results of Mendelian randomization analyses were consistent with these findings.

Positive associations between maternal smoking during pregnancy and offspring smoking initiation remain after adjustment for potential confounding factors and mutual adjustment for partner smoking during pregnancy. These findings are consistent with previous reports from longitudinal studies, which have demonstrated associations between maternal smoking during pregnancy and likelihood of offspring smoking 2,11. However, as discussed previously, it is difficult to infer intrauterine mechanisms from these studies. The similar magnitude of associations observed between partner smoking and offspring smoking initiation observed in our study suggests that these associations are more likely to be due to the shared family environment, either by exposure to parental smoking in childhood and adolescence or by other factors which influence both maternal and offspring smoking. We cannot assess the relative contributions of these in our analyses, but there is evidence in the literature to support a role of familial smoking during childhood in determining offspring smoking initiation 11,14. One study reported a dose–response effect, with number of smoking parents and duration of exposure positively associated with risk of initiation 36.

There was little statistical evidence to support an association between maternal rs1051730 genotype and offspring smoking initiation in pre-pregnancy smokers, but point estimates for late-onset and early-onset regular smokers were consistent with small positive associations of maternal smoking during pregnancy. The Mendelian randomization analysis was based on relatively small numbers, and is therefore unlikely to have been sufficiently powered to detect associations. These results should be taken as exploratory in nature and interpreted in the context of the other evidence presented in this paper. The rs1051730 variant will affect smoking heaviness of the mothers throughout the period that they continue to smoke and so will influence the heaviness of smoking during childhood. Thus, while a null finding in an adequately powered analysis would suggest that there is no *in-utero* effect of pregnancy smoking, positive findings may reflect an influence of maternal smoking throughout childhood.

Shared genetic factors are likely to explain some of the association between maternal smoking and offspring smoking initiation; it has been estimated that about 50% of the variation in smoking behaviour is due to genetic factors 37. However, this should not affect the results of the Mendelian randomization analysis, as it has been shown consistently that variants in the *CHRNA5–CHRNA3–CHRNB4* gene cluster do not associate with smoking initiation 30,38,39. Within this sample, there was no clear evidence for an association between offspring rs1051730 genotype and the smoking initiation classes (data not shown).

One of the key strengths of this paper is the large sample size for the analyses of maternal and partner smoking and dose–response relationships. The use of data from a prospectively assessed cohort ensured that we could be confident we were truly measuring smoking initiation. Use of initial smoking trajectories as a phenotype was also a strength, capturing the complexities of smoking behaviour during adolescence.

One of the limitations of this work is that all the smoking data presented in this paper were obtained from questionnaire data. However, all reports were made at or close to the period of interest. Self-report of smoking behaviour has been demonstrated to be a valid measure of smoking compared to biochemical markers 40 but smoking during pregnancy is likely to be under-reported, particularly by mothers 41. Greater under-reporting of smoking by mothers than partners would serve to attenuate any associations with offspring smoking, but we think that this is unlikely to fully explain the observed similarity between point estimates for maternal and partner smoking.

Furthermore, the sample available for analyses was not fully representative of the whole ALSPAC cohort; offspring with smoking class data tended to be of higher socio-economic position and were less likely to have had parents who smoked during pregnancy. While this is likely to impact upon the prevalence of smoking, there is no obvious reason to think that this would affect associations of maternal and partner smoking differently.

In conclusion, *in-utero* exposure to tobacco may not be an important determinant of offspring smoking initiation and progression to regular smoking. However, we observed positive associations between both maternal and partner smoking during pregnancy and early- and late-onset regular smoking among offspring, suggesting that familial influences may still have an important role to play in intergenerational transmission of smoking behaviour.

### Declaration of interests

None.

## Supporting Information

**Figure S1** Flowchart of the sample available for maternal and partner comparisons. **Figure S2** Flowchart of the sample available for Mendelian randomization analyses. **Table S1** Comparison of analysis sample (*n* = 6484) with complete Avon Longitudinal Study of Parents and Children (ALSPAC) sample. **Table S2** Associations of maternal smoking behaviour during pregnancy with offspring smoking initiation (*n* = 6511).
