# Supplementary material for: Maternal smoking during pregnancy and offspring smoking initiation: assessing the role of intrauterine exposure
Source: Addiction. 2014 Mar 17;109(6):1013–21. doi: 10.1111/add.12514 (PMC4114534; doi:10.1111/add.12514)
Supplement: Supplementary file 1 — Figure S1 Flowchart of the sample available for maternal and partner comparisons. Figure S2 Flowchart of the sample available for Mendelian randomization analyses. Table S1 Comparison of analysis sample (n = 6484) with complete Avon Longitudinal Study of Parents and Children (ALSPAC) sample. Table S2 Associations of maternal smoking behaviour during pregnancy with offspring smoking initiation (n = 6511). [file add-109-1013-sd1.docx]

**Table S1. Comparison of analysis sample (N = 6,484) with complete ALSPAC sample**

|  | Sample included in these analyses | Complete sample excluding siblings | P-value ^1^ |
| --- | --- | --- | --- |
|  | *N (%)* | *N (%)* |  |
| Maternal education  *CSE*  *Vocational*  *O-Level*  *A-Level*  *Degree or above* | 832 (13)  544 (8)  2,245 (35)  1,747 (27)  1,116 (17) | 2,449 (20)  1,194 (10)  4,192 (35)  2,717 (22)  1,559 (13) | <0.001 |
| Housing  *Mortgaged/owned*  *Private rented*  *Council rented*  *Other* | 5,448 (84)  538 (8)  337 (5)  161 (2) | 9,591 (73)  2,094 (16)  953 (7)  468 (4) | <0.001 |
| Parity  *0*  *1*  *2+* | 3,037 (47)  2,311 (36)  1,100 (17) | 5,716 (45)  4,445 (35)  2,592 (20) | <0.001 |
| Mother smoked regularly pre-pregnancy?  *Yes*  *No* | 1,621 (25)  4,863 (75) | 4, 380 (34)  8,573 (66) | <0.001 |
|  | *Mean (SD)* | *Mean (SD)^2^* |  |
| Maternal age (years) | 29.2 (4.5) | 28.0 (5.0) | <0.001 |

1. P values derived from chi square tests for categorical variables and t-tests for continuous variables.
2. N = 13,654.

**Table S2. Associations of maternal smoking behaviour during pregnancy with offspring smoking initiation (N = 6,511)**

|  |  | **Class**  **(percentage membership)^1^** | | | |  |
| --- | --- | --- | --- | --- | --- | --- |
|  |  | **Non-smokers**  **(84%)** | **Experimenters (6%)** | **Late onset**  **(8%)** | **Early onset**  **(2%)** | **P-value^2^** |
|  | **N** | **OR (95% CI)** | **OR (95% CI)** | **OR (95% CI)** | **OR (95% CI)** |  |
| **Did not smoke in pregnancy** | **5,175** | 1 | 1 | 1 | 1 |  |
| **Did not smoke in pregnancy but started after birth** | **100** | 1 | 1.40 (0.72, 2.72) | 1.76 (0.90, 3.45) | 1.86 (0.66, 5.23) |  |
| **Smoked in pregnancy** | **1,236** | 1 | 1.34 (1.07, 1.68) | 1.85 (1.47, 2.32) | 2.99 (2.20, 4.08) | <0.001 |

Adjusted for sex, maternal age, parity, maternal educational attainment, crowding, housing tenure.

1. Percentages in each class of smoking initiation are based on the highest probability of class membership for each individual.
2. P-value for overall association of the exposure with the outcome from Likelihood Ratio Test.

**Figure S1. Flow chart of the sample available for maternal and partner comparisons**

14,451 pregnancies enrolled in ALSPAC

7,109 offspring with smoking initiation data

6,990 with maternal and partner smoking data

6,484 with full covariate data^1^

13,988 offspring alive at 1 year

13,564 unrelated offspring alive at 1 year

6,471 with smoking heaviness and full covariate data^2^

1. For associations of maternal and partner smoking with offspring smoking initiation (Table 2)
2. For comparison of maternal and partner smoking heaviness with offspring smoking initiation (Table 3)

**Figure S2. Flow chart of the sample available for Mendelian randomisation analyses**

14,451 pregnancies enrolled in ALSPAC

7,109 offspring with smoking initiation data

4,434 with maternal rs1051730 genotype

4,192 with mothers of European ancestry

13,988 offspring alive at 1 year

13,564 unrelated offspring alive at 1 year

4,153 with maternal pregnancy smoking data

3,133 pre-pregnancy non smokers^1^

1,020 pre-pregnancy smokers^1^

1. For Mendelian randomisation analysis (Table 4)
